# Randomised trial: survival benefit and safety of adjuvant dose-dense chemotherapy for node-positive breast cancer

**DOI:** 10.1038/sj.bjc.6603085

**Published:** 2006-04-11

**Authors:** S Kümmel, J Krocker, A Kohls, G-P Breitbach, G Morack, M Budner, J-U Blohmer, D Elling

**Affiliations:** 1 Department of Gynecology/Obstetrics, University Medicine Berlin, Charité Campus Mitte, Schumannstr. 20/21, Berlin 10117, Germany; 2 Krankenhaus Lichtenberg, Klinik für Frauenheilkunde, Fanningerstr. 32, Berlin 10365, Germany; 3 Evangelisches Krankenhaus Ludwigsfelde-Teltow, Ludwigsfelde GmbH, Albert-Schweitzer-Strastr. 42-44, Ludwigsfelde 14974, Germany; 4 Staedtisches Klinikum Neunkirchen GmbH, Neunkirchen GmbH, Brunnenstr. 20, Neunkirchen/Saar D-66538, Germany; 5 Helios Klinikum Berlin-Buch, Wiltbergstr. 50, Berlin 13125, Germany; 6 Humaine-Klinikum Bad Saarow, Pieskowerstr. 33, Bad Saarow-Pieskow 15526, Germany

**Keywords:** breast cancer, clinical trial, dose dense, epirubicin, filgrastim, paclitaxel

## Abstract

We evaluated the survival benefit, safety, feasibility, and tolerability of dose-dense (DD) adjuvant chemotherapy with epirubicin and paclitaxel for women with node-positive primary breast cancer. Randomised patients (*n*=216) received DD or conventional-schedule (CS) chemotherapy. Dose-dense regimen patients (*n*=108) received epirubicin 90 mg m^−2^ plus paclitaxel 175 mg m^−2^ in four 14-day cycles, then cyclophosphamide 600 mg m^−2^, methotrexate 40 mg m^−2^, and fluorouracil 600 mg m^−2^ (CMF 600/40/600) in three 14-day cycles, plus filgrastim 5 *μ*g kg day^−1^ as growth support in every cycle. Conventional-schedule regimen patients (*n*=108) received epirubicin 90 mg m^−2^ plus cyclophosphamide 600 mg m^−2^ in four 21-day cycles, then CMF 600/40/600 in three 21-day cycles, plus filgrastim if required. After a median follow-up of 38.4 months, 71 patients (33%) relapsed or died: DD, 33 patients (15 deaths); CS, 38 patients (22 deaths). Dose dense showed a trend for improved disease-free survival (DFS) and overall survival (OS). Four-year rates of DFS and OS were 64 and 85% for DD, and 58 and 75% for CS. All seven cycles were administered to 208 patients (96%). Rates of cycle delay, discontinuation, dose reduction, and adverse events were similar in both groups. Dose-dense sequential chemotherapy with epirubicin/paclitaxel then CMF, supported by filgrastim, is safe and improves survival for patients with node-positive breast cancer.

Dose-dense (DD) adjuvant chemotherapy regimens for node-positive breast cancer limit the opportunity for the regrowth of cancer cells between chemotherapy cycles and improve disease-free survival (DFS) and overall survival (OS) compared with conventional treatment regimens (Wood *et al*, 1994; Budman *et al*, 1998; Citron *et al*, 2003). For breast cancer tumours that are resistant to conventional therapy with cyclophosphamide, methotrexate, and 5-fluorouracil (CMF), anthracyclines (e.g., doxorubicin or epirubicin) and taxanes (e.g., paclitaxel or docetaxel) are active and increase survival for many patients (Glück, 2001).

Anthracyclines and taxanes have different mechanisms of action, little overlap in haemotoxicity, and limited crossresistance (Glück, 2001). In clinical studies, sequential administration of doxorubicin followed by CMF and paclitaxel in combination with doxorubicin improved survival compared to CMF alone (Bonadonna, 1996). Epirubicin provides comparable response rates to those of doxorubicin, with reduced cardiotoxicity at equivalent doses (Lück *et al*, 1996; Glück, 2001). The survival advantages of epirubicin have been confirmed in several clinical trials, including 7- and 10-year studies (Early Breast Cancer Trialists' Collaborative Group, 1998; Levine *et al*, 1998; Fumoleau *et al*, 2003a, 2003b; Poole *et al*, 2003; Bonneterre *et al*, 2005). Recent evidence suggests that adding taxanes to anthracycline regimens may further improve patient survival (Henderson *et al*, 2003; Roché *et al*, 2004; Martin *et al*, 2005).

Using anthracyclines and taxanes in a DD schedule of sequential combination chemotherapy has been shown to confer a survival benefit compared to conventional regimens (Glück, 2001; Citron *et al*, 2003; Möbus *et al*, 2004). However, DD therapy carries an increased risk of febrile neutropenia, a potentially fatal complication for up to 10% of patients. Neutropenia also may increase the risk and cost of treatment and result in dose reduction or delay of planned chemotherapy (Ozer *et al*, 2000). Studies of dose-intense regimens demonstrate that delay in the administration of planned chemotherapy or reduction of doses may be associated with decreased DFS and OS (Bonadonna *et al*, 1995; Budman *et al*, 1998). Growth factor support in DD chemotherapy regimens can reduce the incidence of neutropenic complications, thereby facilitating the administration of a high percentage of chemotherapy doses as planned (Bonadonna, 1996). Because delivery of planned doses of chemotherapy is associated with improved survival, DD regimens often include growth factor support (Bonadonna, 1996; Link *et al*, 2001; Citron *et al*, 2003; Möbus *et al*, 2004).

Our prospective, randomised multicentre clinical study compares the rates of DFS and OS associated with two adjuvant treatment regimens for primary breast cancer in patients with ⩾4 positive lymph nodes: epirubicin and paclitaxel followed sequentially by CMF in a DD schedule, *vs* a standard regimen of epirubicin and cyclophosphamide followed sequentially by CMF in a conventional schedule. The feasibility and tolerability of the regimens are also compared. Growth factor support was provided in all cycles to all patients in the DD group, and as needed to patients in the conventional-schedule (CS) group. Early feasibility and toxicity data have been reported previously (Elling *et al*, 2000); this interim report describes DFS, OS, safety, feasibility, and tolerability after an average of 38.4 months of follow-up.

## PATIENTS AND METHODS

### Study population

This study was conducted in accordance with the ethical principles defined in the Declaration of Helsinki of the World Medical Association. The protocol was approved by each institution's ethics committee, and each enrolled patient provided written informed consent. A total of 231 female patients were enrolled in the study at 30 centres in Germany between July 1996 and December 2000. Patients had primary resected, histologically confirmed breast cancer, stage I, II, or III (Sobin *et al*, 1997). Surgical procedures, performed ⩽15 days before randomisation, included R0 resection and axillary extirpation (levels I to II obligatory, or level III depending on the clinical situation). Patients had ⩾4 positive axillary lymph nodes, no distant metastases, an Eastern Cooperative Oncology Group (ECOG) performance status <2, adequate organ function, and no previous chemotherapy or radiotherapy. Exclusion criteria included leucocytes <3.5 × 10^9^ l^−1^ or platelets <100 × 10^9^ l^−1^. One patient with an ECOG performance status of 3 was admitted to the study in violation of the protocol; this patient was included in the analysis.

### Study design

This multicentre, randomised, open-label Phase 3 adjuvant therapy optimisation study evaluates a DD sequential chemotherapy regimen administered after mastectomy or breast-conserving surgery. The primary end point of the study was the rate of DFS of patients receiving the two sequential regimens. The secondary end points were the rates of OS in the two groups, the incidence of chemotherapy postponement or dose reduction, and the safety and tolerability of the regimen. The study also examined factors that may affect DFS, including hormone receptor status, number of positive lymph nodes, tumour status, malignancy grade, age, haemoglobin values, and delays in chemotherapy administration.

**Figure 1:**
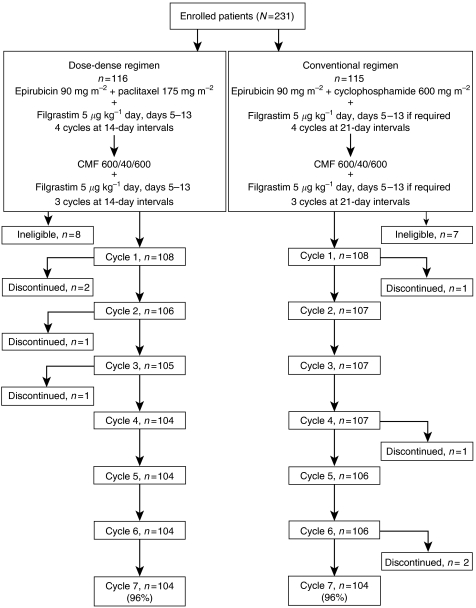
Study design and patient disposition.

Eligible patients were randomised in permuted blocks, stratified by centre, to one of the two chemotherapy treatment groups (DD-schedule group or CS group) using a computer-generated randomisation list (Figure 1). The DD-schedule group received treatment with four cycles of epirubicin and paclitaxel at 14-day intervals, followed by three cycles of CMF at 14-day intervals. All patients in the DD-schedule group received filgrastim in each cycle, administered at a dose of 5 *μ*g kg day^−1^ beginning on day 5 and continuing until either day 13 or leucocyte counts reached >10 × 10^9^ l^−1^. The CS group received a conventional sequential regimen: four cycles of epirubicin and cyclophosphamide at 21-day intervals followed by three cycles of CMF at 21-day intervals. Patients in the CS group were treated with filgrastim in subsequent cycles if they experienced any of the following events during a cycle: absolute neutrophil count (ANC) <0.5 × 10^9^ l^−1^ for more than 2 days, a fever of unknown origin (>38.5°C), infection, or the interval between cycles of chemotherapy prolonged by more than 3 days because of ANC ⩽2.5 × 10^9^ l^−1^. Assigned chemotherapy regimens were initiated no later than 21 days after surgery (tumour resection or mastectomy) if patients met the study entry criteria. Patients were required to exhibit haematologic recovery (ANC ⩾1.0 × 10^9^ l^−1^ and platelets ⩾50 × 10^9^ l^−1^) on the scheduled start day for the next cycle before receiving chemotherapy. The initial postponement was 3 days, followed by a repeated measurement and another postponement, if required, of 3 to 4 days, if haematologic recovery had not occurred.

The treatment phase of the study ended when patients achieved haematologic recovery after the last administration of chemotherapy in accordance with protocol. Patients were followed for up to 5 years after inclusion in the study with regularly scheduled visits.

### Treatment procedures

At baseline and at the end of the study, patients underwent physical examinations, including the measurement of baseline characteristics, disease staging, and performance status, as well as thoracic radiographs, mammography of the contralateral breast, and abdominal sonography. Left ventricular ejection fraction was measured by electrocardiogram or echocardiogram. Other assessments were performed at the investigator's discretion. Complete blood counts (CBC) and other appropriate laboratory tests were also performed. A CBC was obtained twice weekly during treatment. During the period of expected postchemotherapy neutrophil count nadir, a CBC was taken every 2 days. Electrolytes, creatinine, and C-reactive protein measurements were taken weekly.

Each group received two sequences of chemotherapy. The DD-schedule group began the first sequence of epirubicin 90 mg m^−2^ by short intravenous (i.v.) infusion, followed by a 3-h i.v. infusion of paclitaxel 175 mg m^−2^ on day 1, repeated for four cycles at a planned interval of 14 days. In the second sequence of treatment, DD-schedule group patients received three cycles of cyclophosphamide 600 mg m^−2^, methotrexate 40 mg m^−2^, and 5-fluorouracil 600 mg m^−2^ (CMF 600/40/600) by short i.v. infusion at a planned interval of 14 days. The DD-schedule group also received filgrastim 5 *μ*g kg day^−1^ in each cycle during both sequences of chemotherapy. For the CS group, the first chemotherapy sequence consisted of a short i.v. infusion of epirubicin 90 mg m^−2^ and cyclophosphamide 600 mg m^−2^, repeated for four cycles at a planned interval of 21 days. In the second sequence, the CS group received three cycles of CMF 600/40/600 by short i.v. infusion at a planned interval of 21 days. Patients in the CS group could have received filgrastim if required.

Patients receiving paclitaxel received appropriate premedication before administration of paclitaxel. Supportive medications were administered at the investigator's discretion. Patients who experienced febrile neutropenia were treated according to the standard operating procedures of each centre. Patients with a fever ⩾38°C who had not received blood products received antibiotic therapy. Empirical antibiotic therapy in patients with febrile neutropenia without a positive blood culture was discontinued after the patient had been afebrile for 72 h, provided the neutropenia was resolved. Platelet transfusions were given when a patient's platelet count fell to 15 × 10^9^ l^−1^ or below. Erythrocyte concentrates were given in accordance with standard practice at the investigator's institution. All supportive measures, treatments for infection, transfusions, and administrations of erythrocyte concentrate were documented.

All patients with positive oestrogen or progesterone receptor status received tamoxifen 20 mg day^−1^ for 5 years. All patients who had undergone breast-conserving surgery received adjuvant radiotherapy (40–50 Gy) on completion of the chemotherapy.

### Statistical analysis

#### DFS and OS measurements

The trial was designed to detect a difference of 15% (60 *vs* 75%) in the primary end point of DFS after 5 years, with a risk of type 1 error of 5% (one-sided) and a power of approximately 80%, based on the planned sample size of 121 patients in each treatment group. Kaplan–Meier curves were used to analyse DFS and OS. No interim analyses were planned or performed, so multiple analyses were not considered. The randomised and prognostic groups were compared using the log-rank test (Peto *et al*, 1977), and hazard ratios were provided with 95% confidence limits. Unless specifically designated as one-sided, all *P*-values are two-sided. Except for the primary end point, all analyses and statistical tests were performed as descriptive or exploratory.

#### Feasibility, safety, and tolerability

The feasibility of DD chemotherapy in a sequential therapy regimen was measured as the percent of evaluable chemotherapy cycles that were delayed from the planned schedule and the percent of postponed cycles caused by a delay in haematologic recovery. Safety and tolerability were defined in terms of adverse events during treatment and follow-up.

#### Data sets

All data for eligible patients were included in the survival analysis and analysed on the basis of intention-to-treat. All data for patients who received at least one chemotherapy cycle were included in the safety and tolerability analysis, except for two patients for whom haemotoxicity data were not available. Descriptive or exploratory analyses were performed for the results included in this report.

## RESULTS

A total of 231 women aged 26 to 72 years (mean age, 53 years, standard deviation (s.d.) 10 years), 60% of whom were postmenopausal, enrolled at 30 centres between July 1996 and December 2000. They were randomly assigned to one of two schedule groups: 116 patients to DD therapy and 115 patients to a CS regimen. A total of 15 patients were excluded because of ineligibility. In total, 10 patients were ineligible (five in each group) because of a T4 tumour (Sobin *et al*, 1997). The remaining five patients were ineligible for the following reasons: existing metastasis, history of malignant melanoma, previous chemotherapy or radiotherapy, tumour activity with uncertain breast cancer histology, and erroneous chemotherapy administration. The remaining 216 patients entered the study (108 in each treatment group). One patient refused further treatment after cycle 1 (Figure 1).

Of the 231 patients enrolled, 216 (94%) were evaluated for efficacy and safety. Patient and disease characteristics in each treatment group are summarised in Tables 1 and 2. While the original study design excluded patients with >9 positive lymph nodes, the protocol was subsequently amended to permit 48 patients with >9 lymph nodes to be included in the study (26 (24%) in the DD-schedule group, 22 (20%) in the CS group).

### Disease-free survival

At a median follow-up of 38.4 months, 71 patients (33%) experienced a first event of relapse or death, including 33 events (31%) in the DD-schedule group and 38 events (35%) in the CS group.

**Figure 2:**
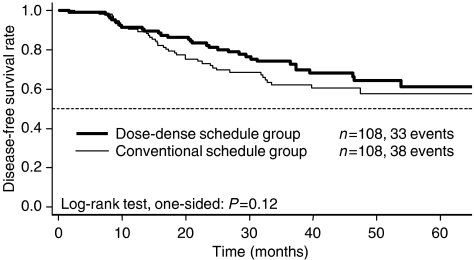
Disease-free survival.

The DFS rate after 2 years was 81% (95% confidence limits (CL), 74–89%) in the DD-schedule group and 72% (64–81%) in the CS group. After 4 years, the DFS rate was 64% (55–76%) in the DD-schedule group and 58% (48–70%) in the CS group. There was trend for DFS in favour of the DD-schedule group, although the difference was not significant (log-rank test, one-sided *P*=0.12) (Figure 2). The hazard ratio, with the DD-schedule group as reference, was 1.32 (0.82 to 2.11) at the time of this analysis. The most common site of disease relapse was bone metastasis (31% of cases), followed by metastasis at local sites, including chest wall, thoracic wall, and axillary and infraclavicular lymph nodes (23%).

### Overall survival

**Figure 3:**
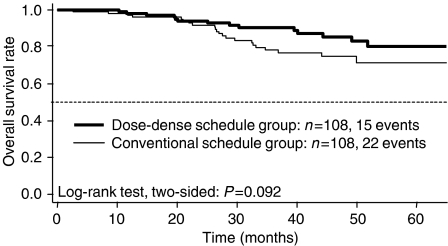
Overall survival.

At a median follow-up duration of 38.4 months, 37 (17%) of 216 patients have died, including 15 patients (14%) in the DD-schedule group and 22 (20%) in the CS group (Figure 3). The OS rate after 2 years was 94% (89–99%) in the DD-schedule group and 92% (87–97%) in the CS group. After 4 years, the OS rate was 85% (78–94%) in the DD-schedule group and 75% (66–85%) in the CS group (Figure 3). The hazard ratio, with the DD-schedule group as reference, was 1.76 (CL 0.91–3.38; log-rang test, two-sided *P*=0.092), suggesting a possible increased risk of death for patients receiving the CS regimen.

### Feasibility (dose reduction or cycle delay)

A total of 1480 cycles of chemotherapy were administered (735 in the DD-schedule group and 745 in the CS group), and 96% of patients in both groups received all seven cycles. The mean (s.d.) cycle length was 14.7 days (3.5) for the DD-schedule group and 21.3 days (1.9) for the CS group.

Commencement of chemotherapy was delayed by more than 1 day in only 10% of DD cycles and 7% of CS cycles. Cycle delays because of delayed haematologic recovery were slightly more common in the DD group (12 cycles, 17%) than in the CS group (six cycles, 12%). Administrative reasons and infection caused most postponements in the other 85% of delayed cycles. No reasons for delayed cycles were reported for two patients.

Doses were reduced in 14 of 1477 evaluable cycles (1%), with no notable differences between groups. (Three cycles were not evaluable because data about dose reductions and delays were missing.) Dose reduction resulting from haematologic toxicity occurred in only one cycle, in the DD-schedule group. In most other cases, doses were reduced because of weight loss resulting in decreased body surface area.

### Safety and tolerability

The safety and tolerability of the two regimens were similar, as assessed by discontinuation and interruption of therapy, laboratory values, and adverse events. The rate of discontinuation was the same (4%) in both treatment groups. One patient in the CS group died during therapy, and one patient in the DD-schedule group refused further treatment after receiving first-cycle therapy. Two patients (2%) in the DD-schedule group and one patient (1%) in the CS group discontinued because of toxicity, including acute hypersensitivity reactions, febrile neutropenia, and fatigue. Three patients (one in the DD-schedule group, two in the CS group) discontinued for other reasons, including withdrawal of consent, uncontrolled diabetes, and infection of a breast wound.

Of 1477 evaluable chemotherapy cycles administered, nine infusions (<1%) had interruptions from 10 min to 24 h (733 cycles in the DD-schedule group, 744 cycles in the CS group). One patient in the DD-schedule group experienced an acute hypersensitivity reaction to paclitaxel, and therapy was discontinued. Reasons for interruption included burning sensations at the infusion site, paravasation, and a defective container.

#### Haematologic toxicity

Filgrastim was administered to all patients in the DD-schedule group in every cycle. In the CS group, filgrastim was administered in 111 of 745 cycles (14.9%), on an as-needed basis. Leukopenia and neutropenia occurred in both treatment groups at similar rates (Table 3). No patients experienced grade 4 anaemia or grade 4 thrombocytopenia. Grade 3 anaemia and grade 3 thrombocytopenia were rare in both groups. Analysis of haematologic toxicity on the basis of cycles yielded similar results.

#### Nonhaematologic toxicity

The most frequently reported nonhaematologic adverse events were alopecia, nausea, and vomiting (Table 3). Fatigue, mucositis, bone pain, and peripheral nervous system (PNS) toxicity were also frequently reported. The incidence of nonhaematologic toxicity was similar in both treatment groups, except for one adverse event commonly associated with paclitaxel and two adverse events commonly associated with filgrastim. Events that occurred more often in the DD-schedule group than the CS group include PNS toxicity (47 *vs* 11%), bone pain (44 *vs* 23%), and arthralgia/myalgia (22 *vs* 15%). Grade 3 cardiotoxicity was extremely rare (one patient in the CS group, none in the DD-schedule group); no patients experienced grade 4 cardiotoxicity.

### Other potential predictors of survival

The effects on DFS of hormone receptor status, menopausal status, number of positive lymph nodes, primary tumour status (T-stage), malignancy grade, age, haemoglobin value, and delays in chemotherapy cycles were also analysed.

#### Oestrogen hormone receptor status (Figure 4)

**Figure 4:**
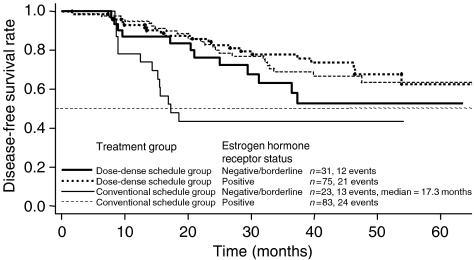
Disease-free survival by treatment group and oestrogen hormone receptor status.

Negative or borderline hormone receptor status had a negative effect on DFS that was statistically significant (two-sided *P*=0.0044). After 2 years, patients with a negative or borderline hormone receptor status had a 62% (50–77%) DFS rate compared with an 82% (76–89%) DFS rate for hormone-receptor-positive patients. After 4 years, the DFS rate for hormone receptor-negative or borderline patients was 48% (35–66%) compared with a 66% (57–75%) DFS rate for hormone-receptor-positive patients. While the data in Figure 4 suggest that patients with negative hormone receptor status profit most from DD therapy, the small sample size warrants a cautious interpretation of this analysis.

#### Menopausal status

Menopausal status had no significant effect on DFS in this study.

#### Number of positive lymph nodes (Figure 5)

**Figure 5:**
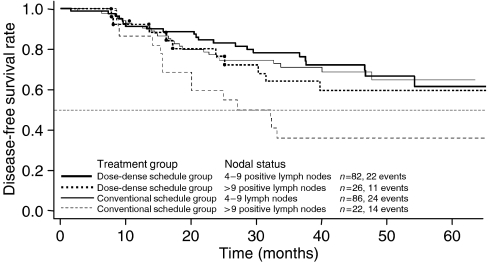
Disease-free survival by treatment group and nodal status.

The prognostic utility of axillary lymph node status in primary breast cancer was confirmed in this study (two-sided *P*=0.017). After 2 years, patients with >9 positive lymph nodes had a DFS rate of 68% (56–83%) compared with 79% (73 to 86%) for patients with 4 to 9 positive lymph nodes. After 4 years, patients with >9 positive lymph nodes had a DFS rate of 48% (35 to 65%) compared with 66% (57 to 75%) for patients with four to nine positive lymph nodes. Patients in the DD-schedule group who had >9 positive lymph nodes had a survival advantage compared to patients with >9 positive nodes in the CS group (Figure 5). As with hormone receptor status, this subgroup analysis should be interpreted with caution because of the small sample size.

#### Tumour status (T-stage)

The stage of the tumour at the time of diagnosis was also a predictor of DFS, with tumours >5 cm showing a clearly less favourable prognosis (two-sided *P*=0.0057). At 2 years, patients with stage T1 tumours had an 85% DFS rate (76–94%) compared with 78% (70–86%) for patients with stage T2 tumours and 64% (50–82%) for patients with stage T3 tumours. After 4 years, DFS was 76% (64–90%) for patients with stage T1 tumours, 62% (52–73%) for patients with stage T2 tumours, and 40% (26–62%) with stage T3 tumours.

#### Malignancy grade

**Figure 6:**
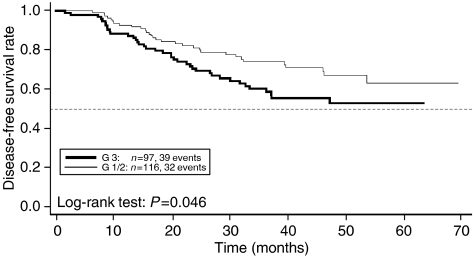
Disease-free survival by malignancy grade.

Malignancy grade was not as strong a predictor of DFS as tumour stage, but differences between malignancy grades were statistically significant (two-sided *P*=0.046). After 2 years, patients with grade G1 or G2 had a DFS rate of 81% (74–89%) compared with 71% (62–81%) for grade G3 malignancies. After 4 years, patients with G1 or G2 malignancies had a DFS rate of 67% (56–78%) compared with 53% (43–66%) for patients with G3 malignancies (Figure 6).

#### Age

As expected, there was a trend toward a less favourable outcome for younger patients, although the trend was not statistically significant. After 2 years, patients ⩾50 years of age had a DFS of 80% (73–87%) compared with 71% (62 to 83%) for patients <50 years. After 4 years, results were similar; patients ⩾50 years of age had a DFS of 63% (54–73%) compared with 57% (45–73%) for patients <50 years.

#### Haemoglobin values

It has been proposed that a low haemoglobin value is associated with a less favourable prognosis. Interim results from this study do not support this hypothesis, either for a baseline haemoglobin <12 g dl^−1^ or for a haemoglobin nadir <11 g dl^−1^ during therapy (data not shown).

#### Chemotherapy cycle delay

Although several studies have shown that a delay in administration of chemotherapy cycles negatively affects DFS, that effect was not significant in this study. The small number of cycle delays ⩾2 days (eight in each treatment group) warrants caution in interpreting these results.

## DISCUSSION

Advances in treatment strategies for node-positive breast cancer include the identification of the chemotherapy agents (including epirubicin and paclitaxel) that are active against tumours resistant to CMF therapy. This study demonstrates that combining epirubicin and paclitaxel followed by CMF in DD adjuvant chemotherapy is feasible, safe, and tolerable for most patients, and that this combination appears to improve OS and DFS in node-positive breast cancer compared with conventional treatment schedules using epirubicin and cyclophosphamide. The use of filgrastim support to prevent neutropenia enhances the safety and tolerability of this regimen. It may also contribute to the rarity of cycle delays in chemotherapy administration in this study.

Discontinuations, cycle delays, and dose reductions were few, which may reflect the lower toxicity of epirubicin and the myeloprotective effects of filgrastim. Peripheral neuropathy, bone pain, and arthralgia/myalgia, which were more common in the DD treatment group, are associated with both paclitaxel and filgrastim; the design of this study does not permit a direct association of these side effects with a single drug.

The benefit of DD therapy for patients with four or more positive axillary nodes is consistent with that seen in Cancer and Leukaemia Group B (CALGB) study 8541 (Wood *et al*, 1994), but the treatment regimen used in our study resulted in somewhat higher DFS rates; in CALGB study 8541 of treatment with cyclophosphamide, doxorubicin, and 5-fluororacil, patients with ⩾4 positive nodes who received high-dose therapy had a DFS rate of 67% after 3 years compared with 62% for a DD therapy schedule and 46% for low-dose therapy. In comparison, 4-year DFS rates in our study were 64% for the DD-schedule group and 58% for the CS group. In CALGB study 8541, patients with ⩾10 positive nodes fared worst, but still did better with DD therapy than with low-dose therapy (44% DFS rate after 3 years with high-dose or DD therapy *vs* 29% for low-dose therapy) (Wood *et al*, 1994). The treatment regimens in our study resulted in a DFS rate of 48% (35–65%) for patients with ⩾10 positive nodes after 4 years, a modest improvement over the CALGB 8541 results.

Results of this study are similar to those reported in the German AGO study, in which estimated 3-year DFS rates were 80% (DD group) and 70% (CS group) for patients with ⩾4 positive lymph nodes receiving regimens of epirubicin, paclitaxel, and cyclophosphamide. (Möbus *et al*, 2004). OS rates in the AGO study were also comparable to those observed in this study (Möbus *et al*, 2004).

In our study, patients with negative hormone receptor status or >9 positive lymph nodes, who have a generally poorer prognosis, benefited from the DD treatment schedule (Figures 4 and 5). At the same time, because patients with >9 lymph nodes fared worse than patients with ⩽9 positive lymph nodes, the inclusion of these patients in the study reduced the OS and the DFS of the total patient population. The potential effect of the number of lymph nodes on survival can be seen by comparing the population in our study with that in the C9741 study (Citron *et al*, 2003). In C9741, 59% of the patients had one to three positive lymph nodes, while all patients in our study had at least four positive lymph nodes. Only 12% of patients in the C9741 study had 10 or more positive nodes compared with 22% of the patients in our study. The 4-year DFS in the C9741 study was 82% for patients receiving DD therapy compared with 64% for patients receiving DD treatment in our study.

In the C9741 study, the DD regimen significantly reduced the incidence of contralateral breast cancer (0.3 *vs* 1.5%, *P*=0.0004) (Citron *et al*, 2003); our study did not confirm that finding, as three patients in each treatment group had contralateral breast cancer. While recent studies of epirubicin-based regimens have shown higher OS and DFS rates than those reported here (Citron *et al*, 2003; Henderson *et al*, 2003; Poole *et al*, 2003), our study is unique in its demonstration of survival benefits for patients with ⩾4 positive axillary lymph nodes.

The results of this prospective, randomised multicentre study provide further evidence that a DD therapy combining anthracyclines with paclitaxel or cyclophosphamide, supported by filgrastim from the first cycle of chemotherapy, can prolong OS and DFS in high-risk patients.

As this is an interim report of a relatively small patient population, caution must be exercised in the interpretation of these results. Fewer patients were originally enrolled than originally planned, potentially reducing the statistical power of the study. Additionally, because of logistic concerns, this regimen may not be feasible in all countries or hospitals. Finally, the risk of leukaemia or myelodysplasia consequent to growth factor therapy must be considered, even though this risk appears to be low and not limited to DD regimens. Hudis *et al* reported an incidence of leukaemia or myelodysplasia ⩽1% in DD and conventional treatment groups after a median 6.5 years of follow-up in the C9741 study. (Ozer *et al*, 2000; Hudis *et al*, 2005). Analysis of mature data at a mean follow-up of 5 years will shed additional light on the selection of an optimum therapy.

## Figures and Tables

**Table 1:** Patient characteristics in each treatment group

	**Dose-dense-schedule group**	**Conventional-schedule group**	**Total**
*Patients randomised (n)*	116	115	231
Not eligible (*n*)	8	7	15
Evaluated for efficacy (*n*)	108	108	216
			
*Age (years) (mean (s.d.))* a	53.3 (9.8)	52.5 (9.8)	52.9 (9.8)
<30 years (*n* (%))	2 (2%)	0 (0%)	2 (1%)
30–39 years (*n* (%))	10(10%)	14 (13%)	24 (11%)
40–49 years (*n* (%))	19 (18%)	30 (28%)	49 (23%)
50–59 years (*n* (%))	48 (46%)	36 (34%)	84 (40%)
60–69 years (*n* (%))	23 (22%)	25 (23%)	48 (23%)
⩾70 years of age	2 (2%)	2 (2%)	4 (2%)
			
*Menopause status at diagnosis* b			
Premenopausal (*n* (%))	42 (39%)	44 (41%)	86 (40%)
Postmenopausal (*n* (%))	65 (61%)	63 (59%)	128 (60%)
			
*Activity status at diagnosis*^c^ *(**Oken et al, 1982**)*			
ECOG 0 (*n* (%))	89 (86%)	87 (84%)	176 (85%)
ECOG 1 (*n* (%))	12 (12%)	16 (15%)	28 (14%)
ECOG 2 (*n* (%))	1 (1%)	1 (1%)	2 (1%)
ECOG 3 (*n* (%))	1 (1%)	0 (0%)	1 (0%)
			
*Patients undergoing surgery (n)*	108	108	216
Breast conserving (*n* (%))	42 (39%)	46 (43%)	88 (41%)
Mastectomy (*n* (%))	66 (61%)	62 (57%)	128 (59%)

ECOG=Eastern Cooperative Oncology Group.

a No date of birth given for five patients.

b No menopause status given for two patients.

c No activity given for nine patients.

**Table 2:** Disease characteristics in each treatment group

	**Dose-dense-schedule group**		**Conventional-schedule group**		
	**Age ⩽50 years (*n*=34)**	**Age >50 years (*n*=70)**	**Age ⩽50 years (*n*=45)**	**Age >50 years (*n*=62)**	**Total** ***n*=211^a^**
*Oestrogen hormone receptor status*^b^ *(n)*	34	70	44	62	210
Positive (*n* (%))	22 (65%)	50 (71%)	32 (73%)	50 (81%)	154 (73%)
Borderline or threshold (*n* (%))	1 (3%)	5 (7%)	1 (2%)	3 (5%)	10 (5%)
Negative (*n* (%))	10 (29%)	14 (20%)	10 (23%)	9 (15%)	43 (20%)
Not determined (*n* (%))	1 (3%)	1 (1%)	1 (2%)	0 (0%)	3 (1%)
					
*Histopathological grade*^c^ *(n)*	34	69	43	62	208
G1 (*n* (%))	1 (3%)	5 (7%)	2 (5%)	4 (6%)	12 (6%)
G2 (*n* (%))	15 (44%)	35 (51%)	16 (37%)	33 (53%)	99 (48%)
G3 (*n* (%))	18 (53%)	29 (42%)	25 (58%)	25 (40%)	97 (47%)
					
*Tumour stage (n)*	34	70	45	62	211
T1 (*n* (%))	8 (24%)	24 (34%)	13 (29%)	15 (24%)	60 (28%)
T2 (*n* (%))	20 (59%)	33 (47%)	26 (58%)	33 (53%)	112 (53%)
T3 (*n* (%))	6 (18%)	13 (19%)	6 (13%)	13 (21%)	38 (18%)
T4 (*n* (%))	0 (0%)	0 (0%)	0 (0%)	0 (0%)	0 (0%)
TX (*n* (%))	0 (0%)	0 (0%)	0 (0%)	1 (2%)	1 (0%)
					
*Nodal stage (n)*	34	70	45	62	211
N1 (*n* (%))	29 (85%)	63 (90%)	43 (96%)	54 (87%)	189 (90%)
N2 (*n* (%))	5 (15%)	6 (9%)	2 (4%)	8 (13%)	21 (10%)
N3 (*n* (%))	0 (0%)	1 (1%)	0 (0%)	0 (0%)	1 (0%)
					
*Lymph node status (n)*	34	70	45	62	211
4–9 involved nodes (*n* (%))	27 (79%)	51 (73%)	40 (89%)	45 (73%)	163 (77%)
>9 involved nodes (*n* (%))	7 (21%)	19 (27%)	5 (11%)	17 (27%)	48 (23%)

a No date of birth given for five patients.

b No hormone receptor status given for one patient.

c No malignancy grades given for three patients.

**Table 3:** Haematologic and nonhaematologic toxicity: highest WHO grade for each patient

	**Toxicity**									
	**Dose-dense-schedule group**					**Conventional-schedule group**				
	**WHO grade^a^ (*n* (%))**					**WHO grade^a^ (*n* (%))**				
	***n***	**0**	**1 or 2**	**3**	**4**	***n***	**0**	**1 or 2**	**3**	**4**
*Haematologic*										
Leukopenia	108	10 (9%)	50 (46%)	40 (37%)	8 (7%)	107	8 (7%)	47 (44%)	46 (43%)	6 (6%)
Neutropenia	77	9 (12%)	20 (26%)	26 (34%)	22 (29%)	82	12 (15%)	17 (21%)	23 (28%)	30 (37%)
Thrombocytopenia	107	82 (77%)	22 (21%)	3 (3%)	0	107	97 (91%)	10 (10%)	0	0
Anaemia	108	14 (13%)	90 (83%)	4 (4%)	0	107	42 (39%)	64 (60%)	1 (1%)	0
										
*Nonhaematologic*										
Alopecia	108	6 (6%)	16 (15%)	86 (80%)	0	107	4 (4%)	14 (13%)	89 (83%)	0
Nausea/vomiting	108	14 (13%)	87 (81%)	7 (6%)	0	107	10 (9%)	85 (79%)	11 (10%)	1 (1%)
Fatigue	108	42 (39%)	58 (54%)	8 (7%)	0	107	52 (48%)	52 (49%)	3 (3%)	0
Mucositis	108	57 (53%)	42 (39%)	9 (8%)	0	107	60 (56%)	45 (42%)	2 (2%)	0
Bone pain	108	60 (56%)	43 (40%)	5 (5%)	0	107	82 (76%)	22 (21%)	2 (2%)	1 (1%)
PNS toxicity	108	57 (53%)	50 (46%)	1 (1%)	0	107	95 (88%)	11 (10%)	1 (1%)	0
Arthralgia/myalgia	108	84 (78%)	20 (19%)	4 (4%)	0	107	91 (84%)	16 (15%)	0	0

PNS=peripheral nervous system; WHO=World Health Organization.

a Because of rounding, percentages may not total 100%.
